# An Adrenal SMARCB1/INI1 Deficient Proximal Epithelioid Sarcoma in a Middle-Aged Female: A Case Report

**DOI:** 10.7759/cureus.29242

**Published:** 2022-09-16

**Authors:** Zachary Brodie, Erin McCartney, Sergio Toledo

**Affiliations:** 1 Internal Medicine, Rocky Vista University College of Osteopathic Medicine, Parker, USA; 2 Pediatrics, Rocky Vista University College of Osteopathic Medicine, Parker, USA; 3 Advanced GI and Bariatric Surgery, Parkview Medical Center, Pueblo, USA

**Keywords:** ini1, proximal epitheloid sarcoma, left adrenalectomy, adrenal mass, smarcb1

## Abstract

Proximal epithelioid sarcomas are rare soft tissue sarcomas that have been documented in a diverse range of presentations. However, there have been few cases describing adrenal presentations. These neoplasms are thought to be driven by a loss of SWItch/sucrose non-fermentable (SWI/SNF)-related matrix-associated actin-dependent regulator of chromatin subfamily B member 1 (SMARCB1), also known as integrase interactor 1 (INI1). SMARCB1/INI1 is a tumor suppressor gene thought to play a role in multiple malignancies with varying degrees of gene expression. Complete loss of SMARCB1/INI1 has most commonly been described in the English scientific literature as malignant rhabdoid tumors of renal origin within pediatric populations and proximal epithelioid sarcomas in adult populations. We describe a case of a primary adrenal proximal epithelioid sarcoma demonstrating complete loss of SMARCB1/INI1 in a middle-aged adult female.

## Introduction

SWItch/sucrose non-fermentable (SWI/SNF)-related matrix-associated actin-dependent regulator of chromatin subfamily B member 1 (SMARCB1), also known as integrase interactor 1 (INI1) is a tumor suppressor gene first discovered in yeast in the 1980s and thought to play a role in chromatin remodeling [1,2]. It has been proposed to be a tumor suppressor gene involved in several signaling pathways [2]. SMARCB1/INI1 deficient tumors are rare and typically classified by the degree of nuclear loss observed on immunohistochemical staining, ranging from partial to complete. Complete loss is most commonly described as malignant rhabdoid tumors (MRT) of renal origin within pediatric populations and as proximal epithelioid sarcomas (ES) in adult populations [1,2]. In children, these neoplasms are thought to be due to germline mutations, typically presenting before the age of three years, and follow a lethal and aggressive course [3]. In adult populations, their origin is less understood. SMARCB1/INI1 deficient proximal ES in adults have been documented in various gastrointestinal, genitourinary, neurological, and cutaneous locations [1,3]. However, very few cases in the literature have described these malignancies arising from the adrenal gland [4-6]. While a loss of SMARCBI/INI1 links these tumors, little is understood regarding other relationships [1]. We describe a case of a malignant primary adrenal proximal epithelioid sarcoma demonstrating complete loss of SMARCB1/INI1 in a middle-aged adult female.

## Case presentation

**Figure 1:**
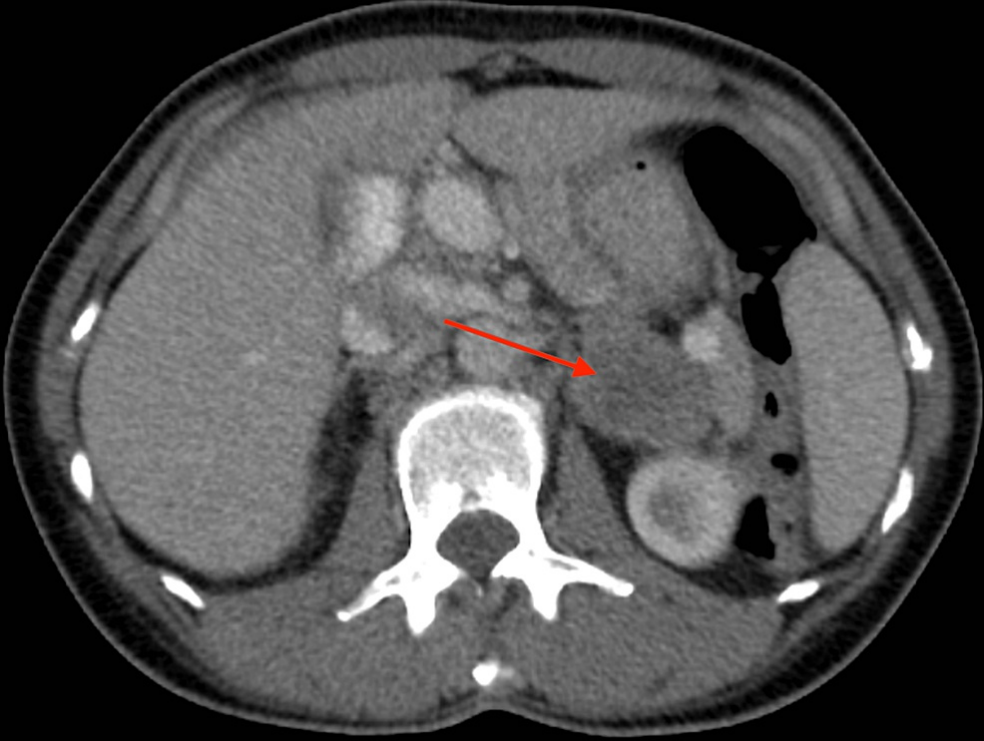
Axial view of non-contrast computed tomography of the abdomen and pelvis demonstrating adrenal mass (arrow).

A 44-year-old female patient with a past medical history significant for gastroesophageal reflux disease and hiatal hernia presented to the emergency department (ED) in May of 2021 with a complaint of left-sided abdominal pain with associated bloating for four months. Physical examination findings were remarkable for tenderness to palpation over the mid and left lateral abdomen with no peritoneal signs. Laboratory values were within normal limits. A non-contrast computed tomography (CT) scan of the abdomen and pelvis was performed as part of the workup, and the findings demonstrated a 4x2.4 cm left-sided adrenal mass (Figure 1). She was discharged from the ED with recommendations to follow-up with a primary care provider for further imaging and workup.

**Figure 2:**
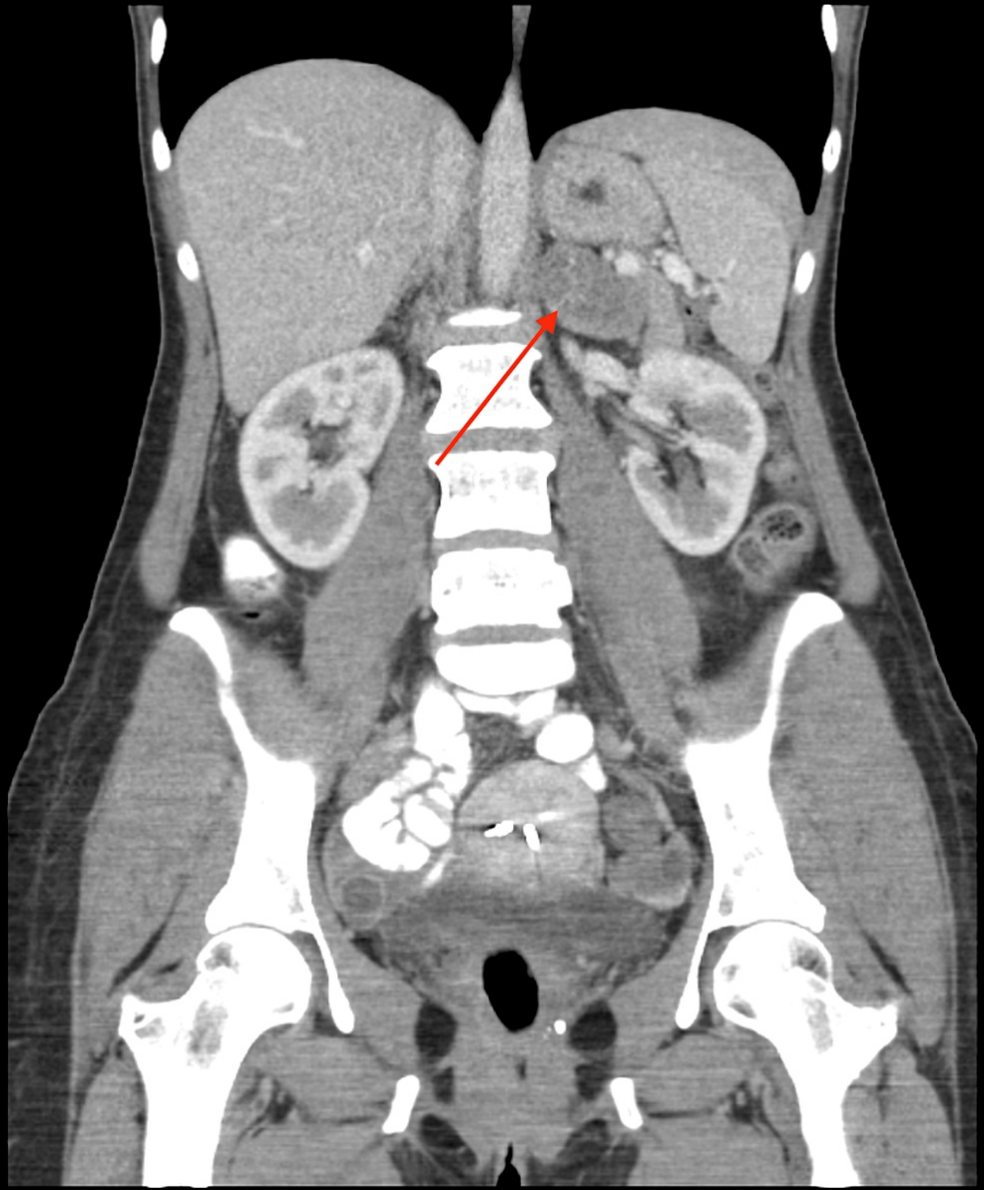
Coronal view of computerized tomography with adrenal protocol demonstrating the left adrenal mass (arrow).

Upon further evaluation by her primary care provider, a biochemical workup was initiated for pheochromocytoma and she was referred to general surgery. The results of the biochemical workup were within normal limits. However, during evaluation by surgery, she reported anxiety, occasional panic attacks, palpitations, and tremors. A follow-up triple-phase, contrasted CT of the abdomen and pelvis with adrenal protocol was done which demonstrated a 4.3 cm left-sided adrenal mass which was indeterminate based on washout (Figure 2). However, surgical removal was recommended due to the size (>4 cm). Due to the patient's reported symptoms, there was a concern for a subclinical pheochromocytoma, and alpha blockade was started prior to surgical intervention. Laparoscopic left adrenalectomy was performed. A 7.2x4.9x2.3 cm adrenal mass with calcifications was removed and sent for further analysis. The surgical course was uncomplicated, and she was discharged on postoperative day one. There were no postoperative complications.

Pathologic examination identified a nodular lesion replacing the entire medulla with a central cavitary area of hemorrhage with clear surgical margins. Microscopic evaluation revealed rhabdoid appearing, sheets and nests of malignant epithelial cells with small nucleoli, high mitotic activity, areas of tumor necrosis, and lymphovascular and perineural invasion. Immunohistochemistry was completed by two laboratories. The pathology results are listed in Table 1. Analysis also demonstrated no rearrangement of Ewing sarcoma RNA-binding protein 1 (ESWR1) but did reveal a complete loss of INI1, consistent with a SMARCB1/INI1-deficient malignant neoplasm.

**Table 1:** Immunohistochemical results as obtained by two laboratories. CDX-2: caudal-related homeobox gene 2, ER: estrogen receptor, GATA3: GATA binding protein 3, Napsin: novel aspartic proteinase of the pepsin family, S100: soluble in 100% saturated ammonium sulfate at neutral pH, CK7: cytokeratin 7, CK20: cytokeratin 20, TTF-1: thyroid transcription factor 1, MART-1: melanoma antigen recognized by T-cells 1, MYO-D1: myogenic differentiation 1, p63: tumor protein 63, CK5: cytokeratin 5, ERG: erythroblast transformation specific related gene, SOX-10: SRY-related HMG-box gene 10, SF1: steroidogenic factor 1, PAX-8: paired box gene 8, WT-1: Wilms tumor 1, HepPar-1: hepatocyte parafin 1

Immunohistochemistry results	Positive	Negative					
	CDX-2	Chromogranin A	S100	MART-1	P63	SF1	Arginase
	Pancytokeratin	ER	CK7	Desmin	CK5	PAX-8	-
	Synaptophysin	GATA3	CK20	MYO-D1	ERG	WT-1	-
	CD34	Napsin	TTF-1	Myogenin	SOX-10	HepPar-1	-

The case was reviewed at tumor board, and a positron emission tomography (PET) scan was recommended to assess for metastasis or recurrent disease. PET CT was chosen due to its high sensitivity (98.5%) and specificity (92%) for assessing malignant adrenal tumors [7]. A PET scan one month postoperatively did not identify any areas of hypermetabolic activity suggesting metastasis or recurrent tumor in the area of the left adrenal gland. Six-month follow-up PET scan was recommended and the patient has had an uneventful follow-up with oncology.

## Discussion

It can be difficult to differentiate between proximal-type ES and MRT as previous literature has reported that both can demonstrate a complete loss of SMARCB1/INI1 expression. MRTs are typically described in pediatric populations, whereas ES are more common in adults [8]. Proximal-type ES have been described most commonly within the pelvic girdle and perineal region [9]. However, some differentiation can be made using immunohistochemical staining. CD34 is positive in half of ES presentations and typically negative in MRT [8,9]. Given the positive staining for CD34 in this case, the tumor is more likely a proximal-type ES rather than MRT. Additionally, immunohistochemistry was negative for S100 and desmin, consistent with proximal ES [9,10].

Proximal ES typically have an aggressive course with poor five-year survival rates. In adult populations, they demonstrate a male predominance [1,10]. Proximal ES have high recurrence rates, reported as high as 77%, and approximately half will have metastatic disease [10]. These malignancies are thought to metastasize via lymphatic spread [10]. If there is metastasis, it will most commonly be to the lungs followed by local lymph nodes [10].

Proximal ES arising from the adrenal gland has been rarely reported. We believe that this is the fourth case described in the literature [4-6]. Compared to previously reported cases, this presentation is consistent with vague abdominal pain and a mass discovered on imaging [4-6]. Non-functional biochemical workup is consistent across published cases. The age of presentation has ranged from 31 to 81 years of age. Of proximal ES arising from the adrenal gland, three out of four cases have been reported in female patients and occurred in the left adrenal gland [4-6]. Half of the adrenal cases had suspected lymph node involvement at resection [4-6]. In both instances, those with lymph node involvement had recurrence or progression of disease after surgical resection [4,5]. Unfortunately, due to such a small number of cases available in the literature, no specific conclusions can be drawn regarding the epidemiology of proximal ES arising in the adrenal gland.

This is most likely a primary tumor rather than metastatic disease. Initial imaging was only apparent for an adrenal mass, and follow-up PET CT postsurgical resection did not demonstrate any areas of increased metabolism. The tumor was positive for CDX-2 which can be a marker of colorectal metastasis [11,12]. However, the rest of the clinical history and microscopic investigation were not consistent with adenocarcinoma, making a metastatic colorectal source less likely. Immunohistochemical staining did not identify any other likely tissue source of potential metastasis including lung, thyroid, breast, or myoepithelial tissue [12]. Additionally, with the typically aggressive nature of SMARCB1/INI1 tumors, a metastatic origin would have likely been apparent on PET CT as increased tumor burden within the lungs.

## Conclusions

Although rare, the aggressive nature of these malignancies necessitates early removal. While rarely associated with adrenal masses, this case highlights the importance of removing non-functional adrenal masses that are larger than 4 cm. Because of the high rate of metastatic disease at presentation, follow-up after diagnosis and surgical removal should include a PET CT to assess tumor burden/metastasis. Follow-up should be consistent with national soft tissue sarcoma guidelines and based on initial tumor location.
